# Associations between brominated flame retardants, including polybrominated diphenyl ethers, and immune responses among women in the California Teachers Study

**DOI:** 10.3389/fepid.2025.1452934

**Published:** 2025-03-19

**Authors:** Emily L. Cauble, Peggy Reynolds, Marta Epeldegui, Priyanthi S. Dassanayake, Larry Magpantay, Daniel Blyakher, Pratima Regmi, Julie Von Behren, Otoniel Martinez-Maza, Debbie Goldberg, Emma S. Spielfogel, James V. Lacey, Sophia S. Wang

**Affiliations:** ^1^ Division of Health Analytics, Beckman Research Institute, City of Hope, Duarte, CA, United States; ^2^ Department of Epidemiology and Biostatistics, University of California San Francisco, San Francisco, CA, United States; ^3^ Department of Obstetrics and Gynecology, David Geffen School of Medicine, University of California Los Angeles, Los Angeles, CA, United States; ^4^ Jonsson Comprehensive Cancer Center, University of California Los Angeles, Los Angeles, CA, United States; ^5^ UCLA AIDS Institute, University of California, Los Angeles, Los Angeles, CA, United States; ^6^ Department of Environmental Medicine and Climate Science, Icahn School of Medicine at Mount Sinai, New York, NY, United States

**Keywords:** brominated flame retardants (BFRs), polybrominated diphenyl ether (PBDE) congeners, cytokines, immune responses, inflammatory markers, women, human population

## Abstract

**Objective:**

To evaluate the associations between brominated flame retardants (BFRs), including polybrominated diphenyl ethers (PBDEs), exposure and circulating immune markers in a subset of women from the California Teachers Study cohort.

**Methods:**

In this cross-sectional study, serum from 813 female participants in the California Teachers Study collected in 2013–2016 were evaluated for 11 BFR congeners and 16 immune markers. Three BFR congeners [BDE153 [2,2′,4,4′,5,5′-Hexabromodiphenyl ether], BDE47 [2,2′,4,4′-Tetrabromodiphenyl ether], PBB153 [2,2′,4,4′,5,5′-Hexabromobiphenyl]] had median levels that were above the level of detection and were further evaluated for associations with circulating immune markers. Odds ratios (OR) and 95% confidence intervals (CI) were calculated by a logistic regression model where BFR congeners (in quartiles) were associated with immune markers (dichotomized as above and below the respective median), adjusted for age and total lipids. Sensitivity analyses were also conducted evaluating BFR congeners as a continuous exposure (per pg/ml).

**Results:**

All participants had at least one of the 11 measured BFR congeners detected in their serum. Increasing levels of BDE47 were associated with elevated levels of BAFF (B-cell activating factor; OR_Quartile 4_ = 1.67, 95% CI = 1.11–2.51), soluble CD27 (sCD27, cluster of differentiation 27; OR_Quartile 4_ = 1.69, 95% CI = 1.12–2.55) and IL6 (interleukin 6; OR_Quartile 4_ = 1.74, 95% CI = 1.13–2.66). Increasing levels of PBB153 were associated with elevated levels of CXCL13 (chemokine ligand 13; OR_Quartile 4_ = 1.55, 95% CI = 1.02–2.35) but inversely associated with sCD27 (OR_Quartile 4_ = 0.57, 95% CI = 0.38–0.87). Results from continuous models of BFR were largely consistent. No associations were observed between BDE153 and any of the immune markers assessed.

**Conclusions:**

Two BFR congeners were statistically associated with altered levels of circulating immune markers involved in B cell activation pathways; replication and further evaluation of these novel associations are warranted. If confirmed, our results add to the current literature regarding possible immune mechanisms by which BFR exposures contribute to immune-related health endpoints and conditions where B cell activation is prominent, including autoimmune conditions.

## 1 Introduction

Brominated flame retardants (BFRs), including polybrominated diphenyl ethers (PBDEs), are chemicals that were first manufactured in the 1970s and used as flame retardants in various materials and products, such as plastic, automobiles and wiring material (1). The use of these chemicals is often associated with commercial mixtures known as pentabromodiphenyl ether (c-pentaBDE), hexabromodiphenyl ether (c-hexaBDE), octabromodiphenyl ether (c-octaBDE), and decabromodiphenyl ether (c-decaBDE). Per the Centers for Disease Control and Prevention (CDC), BFR congener levels are often correlated due to their production and use within commercial mixtures (2); the detected levels indicate cumulative effects of long-term exposures (2). In the early 2000s, the United States Environmental Protection Agency (EPA) released a biomonitoring summary to describe the overall human exposure and potential health risks caused by BFR exposure, resulting in the replacement of these chemicals (e.g., c-pentaBDE, c-octaPBDE, and c-decaBDE) in production (2).

Despite restriction of their use, BFRs remain pervasive in the environment and persistently detected in humans and are of particular concern for aging populations which have the highest accumulated levels during a period of time in which they are likely to face health effects. There are multiple routes of exposure to BFR during one's lifetime, including through inhalation, dermal contact, dietary intake and through contaminated water (3). A cross-sectional study conducted in 2008 within the National Health and Nutrition Examination Survey (NHANES) that assessed BFR levels in 2,062 adult serum samples found that at least one BFR congener was detected in 99% of the samples (4). Similar prevalences demonstrating the pervasiveness of BFR in human populations have been reported in various studies (5, 6).

BFR exposure has been linked to hormone dysregulation, thyroid dysfunction, and altered metabolic activity; it has also been linked to increased risk of developing type 2 diabetes, increased cancer risk, and chronic obstructive pulmonary disease (7–10). Additionally, PBDE congeners have been reported at high values in young children from gestational exposure (3, 11–13). Previous studies have reported that PBDEs are metabolized by cytochrome P450 and form OH-PBDEs, which can bind hormone receptors and thus act as endocrine disrupting chemicals (8). Importantly, endocrine disrupting chemical exposure has been linked to altered immune responses (14). Exposure to BFR congeners has been shown to cause adverse immune responses through multiple posited biological mechanisms: (i) directly through immune cell function, (ii) by disrupting hormonal balance that can result in altered thyroid activity, and (iii) inducing oxidative stress and producing reactive oxygen species (3, 14).

At present, there are sparse population-based studies that have assessed the associations between BFR exposure and circulating immune markers (5). However, there is growing *in vivo* and *in vitro* evidence to support an association (15–19). An *in vivo* study of BDE47 (2,2′,4,4′-Tetrabromodiphenyl ether)-exposed mice over a period of 30 days across varying dosages and found that exposure to BDE47 reduced the production of both interleukin 6 (IL6) and tumor necrosis factor alpha (TNF-α) (16). Similarly, Longo et al. (2019) found reduced levels of interleukin-1 beta (IL-1β), IL6 and TNF-α in an *in vitro* study that exposed human macrophages to various levels of BDE47 (18). There remains inconsistency in study results, however, as Wei et al. (2020) reported a positive association between BDE47 with TNF-α, IL-1β and IL6 in an *in vivo* mouse study (17). Another *in vitro* study reported that penta-BDE and octa-BDE were positively associated with elevated IL6 and interleukin 8 (IL8) expression in human bronchial epithelial cells (19). Of note, because humans are exposed to BFR congeners through a variety of mediums, including direct contact with fire retardant products and through diet, *in vitro/in vivo* studies may not exemplify human exposure and whether results from mechanistic studies will be observed in human populations has yet to be determined.

In the present study, we measured 11 BFR congeners in blood collected between 2013 and 2016 from a cross-sectional sample of 813 female participants in the California Teachers Study and evaluated the association between BFR congeners with 16 circulating immune markers from the same blood collection. Due to the persistent nature and bioaccumulation of BFR congeners, identifying the potential underlying biological mechanism by which BFR may contribute to adverse health effects is of public health significance. Our study population is targeted among women as immune function decreases with age and women notably have higher risk for autoimmune disease development than men; our study thus provides the opportunity to investigate the associations between BFR exposure and immune markers in a particularly susceptible population subset that may be more at risk for adverse immune-specific health outcomes (20). We hypothesized that individuals with higher circulating levels of BFR congeners would also have higher levels of circulating immune markers.

## 2 Methods

### 2.1 Study population

The California Teachers Study (CTS) is a prospective cohort study that began in 1995 and is comprised of women who were active or recently retired public school professionals. The study population has been previously described, and the study has been approved by the Institutional Review Boards of participating institutions, including City of Hope National Medical Center (21). For this cross-sectional study, participants were selected based on the completion of the coinciding follow-up questionnaire (Questionnaire 5; administered 2012–2015; *N* = 61,984) with the CTS biobanking study (2013–2016; *N* = 13,888); of the eligible population, 814 participants who were cancer-free at the time of blood collection were selected for measurement of BFR congeners, of which data from 813 participants were analyzable. These individuals were initially selected for an immune profiling project where immune markers were assessed as part of a larger study on sleep characteristics.

### 2.2 Cytokine and immune marker measurements

Methods and results for measuring cytokine levels in serum for the participants in this study are previously described (22, 23). Briefly, all immune marker measurements were conducted at the University of California, Los Angeles (M.E.) by multiplexed immunometric assays (Luminex platform) and a Bioplex 200 system (Bio-Rad) using two Luminex panels (R&D Systems): Soluble Receptor Human Panel (soluble receptors and chemokines) and Human Biomarker A Panel (human inflammatory cytokines) (22). The Soluble Receptor Human Panel included the detection of BAFF (B-cell activating factor), CXCL13 (chemokine ligand 13), CD14 (cluster of differentiation 14), sCD27 (soluble cluster of differentiation 27), GP130 (glycoprotein 130), IL-2Rα (interleukin 2 receptor subunit alpha), IL6Rα (interleukin 6 receptor subunit alpha), and TNFR2 (tumor necrosis factor receptor 2). The Human Biomarker A Panel included the detection of IL1β (interleukin-1 beta), IL2 (interleukin-2), IL4 (interleukin-4), IL6 (interleukin-6), IL8 (interleukin-8), IL10 (interleukin-10), IFN-γ (interferon gamma), and TNF-α (Tumor necrosis factor alpha) (22). The serum samples used in the assays were not previously thawed and stored at −80 for 6–9 years. As previously described, all specimens were handled in a single batch and 10% of quality control samples were interspersed (*n* = 83) (22). Another 10% (*n* = 83) of participants were tested in duplicate permitting calculation of coefficient of variations which ranged from 5.17% to 27.74%, with the majority <10% (22).

### 2.3 BFR measurements

A total of 11 BFR congeners were assessed at the Mount Sinai Targeted Analysis Laboratory Hub: 2,2′,4-Tribromodiphenyl ether (BDE17), 2,4,4′-Tribromodiphenyl ether (BDE28), 2,2′,4,4′-Tetrabromodiphenyl ether (BDE47), 2,3′,4,4′-Tetrabromodiphenyl ether (BDE66), 2,2′,3,4,4′-Pentabromodiphenyl ether (BDE85), 2,2′,4,4′,5-Pentabromodiphenyl ether (BDE99), 2,2′,4,4′,6-Pentabromodiphenyl ether (BDE100), 2,2′,4,4′,5,5′-Hexabromodiphenyl ether (BDE153), 2,2′,4,4′,5,6′-Hexabromodiphenyl ether (BDE154), 2,2′,3,4,4′,5′,6-Heptabromodiphenyl ether (BDE183) and 2,2′,4,4′,5,5′-Hexabromobiphenyl (PBB153). BFR congeners were measured at the Senator Frank R. Lautenberg Environmental Health Sciences Laboratory at the Icahn School of Medicine at Mount Sinai using 500ul serum samples. The analytical method was based on published methods by Hovander et al. (2000) (24), EPA (25) and CDC (26) with modifications. Briefly, samples were spiked with 13C isotopically labeled internal standard prior to extraction. Liquid-liquid extraction was automated using a liquid handler (epMotion 5073; Eppendorf, Hauppauge, NY). Hexane and dichloromethane mixture (4:1 v/v) was use for the extraction. Co-extracted impurities were removed from the extract using multi sorbent multi layered cleanup columns. Purified extracts were evaporated under a gentle stream of nitrogen and reconstituted in hexane for instrumental analysis. The GC-MS/MS analysis was performed using an Agilent 7010B triple quadrupole mass spectrometer coupled with Agilent 8890 gas chromatograph (Agilent Technologies, Wilmington, DE). Chromatographic separation was achieved by using a 30 m BD-XLB (0.25 mm ID×0.1 µm film thickness) capillary column with a helium carrier gas flow rate set at 1.4 ml/min. The MS was operated in the EI mode at −70 ev. Multiple reaction monitoring (MRM) was utilized during data acquisition. Isotope dilution internal standard calibration method was used for the quantitative analysis. NIST standard reference material SRM 1958 (Organic Contaminants in Fortified Human Serum), spiked and un-spiked matrix, procedural and instrumental blanks were analyzed alongside samples as quality assurance measures. Within the duration of sample analysis, the analytical lab successfully completed two rounds of AMAP proficiency testing for persistent organics analyses in human serum. All BFR measurements were processed within a 28-hour time period and exhibited low pre-analytical variability due to the adherence to previously established standardized procedures (over 99.5% protocol compliance resulted in minimal pre-processing sample errors). Serum triglycerides and total cholesterol were analyzed at the Clinical Chemistry Laboratory of Mount Sinai Hospital (1425 Madison Ave. New York NY 10029). One batch of lipid analysis consisted of 40 study samples and 2 blinded QC samples (HHEAR Pool C and D) for a total of 22 batches. Additionally, all analyses occurred on the same day. According to previous studies of the California Teachers Study that assessed PBDE concentration, total lipid measurements were calculated by aggregating log-transformed triglycerides and cholesterol measurements per participant (27). The BFR congener dataset presented in this study can be found in the online Human Health Exposure Analysis Resource data repository (https://hhearprogram.org/data-services).

### 2.4 Covariates

Participant characteristics and potential confounders were assessed at baseline, including age (40–49, 50–59, 60–69, or 70+ years), race/ethnicity (non-Hispanic White or other), socioeconomic status (SES; in quartiles determined by occupation, education, and income), rural/urban residence (rural, town, city, metropolitan suburban, or metropolitan urban; according to the 1990 census block groups), as well as updated information ascertained during Questionnaire 5, including weight [for which updated body mass index (BMI) was calculated; BMI: 15–24, 25–29, or 30+  kg/m^2^], physical activity (moderate/strenuous: 0–2.37, 2.38–5.88, 5.88+  hrs/week), nonsteroidal anti-inflammatory drug (NSAID) use (none/1/week or >1/week), and statin use (none or >1/week) (22).

### 2.5 Statistical analysis

Immune marker and BFR congener measurements were characterized for percent detection, medians, and ranges and both log-transformed for univariate and multivariable analyses to normalize the data distribution. Statistical models were constructed for the three BFR congeners that had detectable median values (BDE153, BDE47, and PBB153), which is consistent with other population studies (4, 5, 28, 29). The associations between BFR congeners (defined as the exposures) and immune markers/pathways (defined as the outcomes) were assessed by both linear and logistic regression, allowing evaluation of exposure (BFR congeners) as continuous and categorical exposure variables. For logistic regression models, BFR congeners were evaluated as quartiles so that no threshold effect would be missed, and immune markers were dichotomized as above and below the respective median value based on methods reported in the literature (22, 27). Immune markers that had <50% detection frequency (IFN-γ, IL2, and IL4) were dichotomized as detectable vs. non-detectable. Multivariable models included adjustments for age (categorical), BMI (categorical), and total serum lipids (log-transformed triglycerides and cholesterol). Final multivariable models for BDE47 also included adjustment for PBB153, and vice versa. Multivariable models were also conducted stratified by key participant characteristics (age, BMI, statin use and diabetes status) to ensure that associations of the BFR exposures and immune marker outcomes did not differ by key covariates.

Immune marker pathways were defined as previously described (22) and assessed for association with BFR congeners, with individual cytokines first dichotomized as above or below the respective median and subsequently categorized as: (1) Pro-inflammatory/macrophage activation: elevated levels of TNF-α, TNFR2, IL6, IL1β, IL8, IL6Rα, IL10, and CD14, (2) Th1: elevated IFN-γ and decreased IL10 and IL-4, (3) B-cell activation: elevated levels of BAFF, IL10, IL4, IL6, sCD27, CXCL13, and (4) T-cell activation: elevated levels of IL2, IL2R*α*, IFN-γ, IL4, IL6. Individuals who had specified immune marker that was above its respective median were considered to be “positive” for that immune marker, which further contributed to their potential categorization in the corresponding immune pathway. An individual was considered “positive” for a specified immune pathway if the number of immune markers identified as “positive” was above the median overall. We conducted pathway-based analyses in order to identify if there were associations (beyond those individually assessed with the immune markers) between BFR congener exposure and broad immune pathways that could provide more insight into specific biological pathways. All data analyses were performed utilizing the California Teachers Study Researcher Platform (30). Statistical analyses were performed using SAS 9.4 (SAS Institute Inc., Cary, NC), and figures were created using GraphPad Prism 9.5.0.

## 3 Results

### 3.1 Study participant characteristics

Select study participant characteristics are shown in Table 1. In general, our study participants were younger (44.8% are 60–69 years old) than the overall California Teachers Study population that completed the follow-up questionnaire (40.4% are 70+ years old) but reflected the sampling of participants that participated in the cohort's biobanking study. Our study participants were also more racially diverse than the overall cohort (79.2% non-Hispanic White vs. 87.6%).

**Table 1:** Distribution of demographic and host characteristics among a subset of 813 participants in the California Teachers Study cohort with simultaneous measurements of serum immune markers and brominated flame retardants (BFR) in 2013–2016.

Characteristic	Cohort with completed Q5 questionnaire (*n* = 61,984^a^)		Cohort with completed Q5 questionnaire and blood sample collected (*n* = 13,888^a^)		Cohort with cytokine and BFR measurements (*n* = 813^a^)	
	*N*	%	*N*	%	*N*	%
Age (in years)						
40–49	4,393	7.1%	1,333	9.6%	102	12.5%
50–59	10,244	16.5%	3,452	24.9%	233	28.7%
60–69	22,272	35.9%	6,586	47.4%	364	44.8%
70+	25,044	40.4%	2,517	18.1%	114	14.0%
Race						
Non-hispanic white	54,294	87.6%	12,391	89.2%	644	79.2%
Other	7,690	12.4%	1,497	10.8%	169	20.8%
Socioeconomic status (SES)						
Quartile 1	2,376	3.9%	457	3.3%	28	3.5%
Quartile 2	9,867	16.2%	2,155	15.7%	116	14.4%
Quartile 3	19,987	32.8%	4,607	33.5%	271	33.6%
Quartile 4	28,755	47.2%	6,533	47.5%	391	48.5%
Body mass index (BMI)						
15–24	29,196	49.7%	6,744	50.1%	378	47.8%
25–29	17,967	30.6%	4,056	30.1%	238	30.1%
30+	11,580	19.7%	2,672	19.8%	174	22.0%
Physical Activity (hr/week)						
0–2.37	20,947	34.3%	3,879	28.2%	279	34.4%
2.38–5.88	20,136	33.0%	4,771	34.6%	265	32.6%
5.88+	20,037	32.8%	5,132	37.2%	268	33.0%
NSAID use						
None or 1/week	24,616	42.3%	5,784	43.3%	357	45.1%
>1/week	33,630	57.7%	7,581	56.7%	434	54.9%
Diabetes						
No	56,246	91.6%	12,891	93.4%	734	90.6%
Yes	5,128	8.4%	907	6.6%	76	9.4%
Statin use						
None	42,336	69.9%	10,188	74.4%	594	73.4%
>1/week	18,223	30.1%	3,513	25.6%	215	26.6%
Rural/urban residence						
Rural	8,813	14.4%	1,782	13.0%	55	6.8%
Town	2,032	3.3%	423	3.1%	12	1.5%
City	10,905	17.9%	2,627	19.1%	59	7.3%
Suburban	33,386	54.7%	7,586	55.1%	578	71.7%
Urban	5,874	9.6%	1,342	9.8%	102	12.7%

^a^ Sample sizes may not sum to the total sample size per group due to unknown covariate measures.

### 3.2 BFR congener and immune marker measurements

Descriptive statistics (median and range) for the 11 BFR congeners are shown in Supplementary Table S1. Three congeners with median levels that were above the limit of detection (BDE153 = 33.4 pg/ml, BDE47 = 70.3 pg/ml, and PBB153 = 18.6 pg/ml) were evaluated further for associations with immune markers and immune pathways. The remaining BFR congeners (i.e., BDE100, BDE154, BDE17, BDE183, BDE28, BDE74, BDE66, BDE85, and BDE99) had medians that were below the limit of detection value and were not further evaluated for associations with immune markers. Descriptive statistics for the 16 immune markers are shown in Supplementary Table S2. BFR congener correlation coefficients (via Spearman's correlation coefficients) are presented in Supplementary Table S3. Of the congeners included in further analyses (BDE153, BDE47, and PBB153), there were no notable correlations. The correlations did not change by population subsets (age, BMI, statin use, diabetes use) (data not shown).

### 3.3 Univariate models

Participant characteristics shown previously to be associated with circulating immune markers were evaluated with BDE153, BDE47 and PBB153 (4, 29, 31, 32), and the results are displayed in Table 2. While higher BMI was associated with increasing levels of BDE47 (30+ kg/m^2^: OR = 1.83, 95% CI = 1.33–2.54), higher BMI was associated with lower levels of both BDE153 (BMI 30+  kg/m^2^ OR = 0.61, 95% CI = 0.44–0.84) and PBB153 (BMI 30+ kg/m^2^ OR = 0.54, 95% CI = 0.39–0.75). Individuals who reported having diabetes also had higher levels of BDE47 (OR = 1.65, 95% CI = 1.07–2.52). Regular NSAID use was associated with lower levels of BDE47 (OR = 0.71, 95% CI = 0.55–0.92) and PBB153 (OR = 0.77, 95% CI = 0.60–0.99). Statin use >1/week was also associated with higher levels of BDE47 (OR = 1.38, 95% CI = 1.04–1.83) but not associated with BDE153 or PBB153. Finally, non-White race was also associated with higher levels of BDE47 (OR = 1.37, 95% CI = 1.01–1.86).

**Table 2:** Associations between participant characteristics and each quartile of log-transformed BFR congener (BDE153, BDE47 and PBB153) and among the subset of 813^a^ participants in the California Teachers Study cohort who had concurrent immune marker and BFR congener measurements from blood collected from 2013–2016. Associations were adjusted for total lipids.

Characteristic	BDE153					BDE47					PBB153				
	Q1	Q2	Q3	Q4	OR (95% CI)	Q1	Q2	Q3	Q4	OR (95% CI)	Q1	Q2	Q3	Q4	OR (95% CI)
Race															
Non-hispanic white	173	152	163	156	1.00 (Reference)	159	158	166	161	1.00 (Reference)	158	163	163	160	1.00 (Reference)
Other	34	49	39	47	0.89 (0.66–1.20)	43	46	39	41	1.37 (1.01–1.86)	44	39	43	43	0.92 (0.68–1.24)
Age (years)															
40–49	26	24	18	34	1.00 (Reference)	23	22	31	26	1.00 (Reference)	36	21	20	25	1.00 (Reference)
50–59	57	53	62	61	1.00 (0.66–1.54)	63	55	40	75	1.03 (0.67–1.57)	46	56	59	72	0.81 (0.53–1.24)
60–69	96	99	93	76	0.82 (0.55–1.23)	88	102	98	76	0.93 (0.62–1.39)	84	99	98	83	0.99 (0.66–1.49)
70+	28	25	29	32	0.78 (0.48–1.27)	28	25	36	25	0.82 (0.51–1.33)	36	26	29	23	1.40 (0.86–2.27)
Socioeconomic status (SES)^a^															
Quartile 1	5	8	5	10	1.00 (Reference)	7	7	5	9	1.00 (Reference)	10	7	7	4	1.00 (Reference)
Quartile 2	21	30	27	38	1.50 (0.71–3.14)	30	28	23	35	1.15 (0.55–2.42)	27	26	33	30	0.81 (0.38–1.70)
Quartile 3	63	66	73	69	1.10 (0.55–2.21)	56	77	71	67	0.97 (0.48–1.95)	62	77	67	65	0.74 (0.37–1.49)
Quartile 4	116	95	95	85	1.00 (0.50–1.99)	106	91	104	90	0.63 (0.32–1.25)	102	89	96	104	0.63 (0.31–1.25)
Diabetes^a^															
No	178	186	187	183	1.00 (Reference)	177	184	185	188	1.00 (Reference)	183	180	186	185	1.00 (Reference)
Yes	27	15	14	20	0.86 (0.56–1.31)	24	19	20	13	1.65 (1.07–2.52)	19	20	20	17	0.77 (0.50–1.18)
Body mass index (BMI, kg/m^2^)^a^															
15–24	96	97	97	88	1.00 (Reference)	90	99	95	94	1.00 (Reference)	92	102	88	96	1.00 (Reference)
25–29	57	58	57	66	0.94 (0.70–1.26)	65	56	62	55	1.48 (1.10–1.99)	58	54	57	69	0.81 (0.61–1.09)
30+	51	38	42	43	0.61 (0.44–0.84)	42	44	43	45	1.83 (1.33–2.54)	46	43	53	32	0.54 (0.39–0.75)
Physical activity (hr/week)^a^															
0–2.37	57	73	72	77	1.00 (Reference)	63	63	76	77	1.00 (Reference)	74	67	69	69	1.00 (Reference)
2.38–5.88	83	64	60	58	0.91 (0.68–1.24)	75	67	67	56	0.92 (0.68–1.25)	67	75	69	54	0.82 (0.61–1.12)
5.88+	66	64	70	68	1.16 (0.86–1.57)	63	74	62	69	0.86 (0.64–1.17)	61	60	68	79	1.03 (0.76–1.40)
Rural/urban residence^a^															
Rural	12	14	13	16	1.00 (Reference)	15	13	13	14	1.00 (Reference)	12	11	13	19	1.00 (Reference)
Town	3	3	1	5	1.00 (0.33–3.09)	5	3	1	3	1.95 (0.63–6.06)	3	1	5	3	0.97 (0.32–2.99)
City	15	13	16	15	0.94 (0.49–1.83)	16	13	17	13	0.83 (0.43–1.61)	10	20	14	15	0.81 (0.42–1.57)
Suburban	153	145	145	135	0.61 (0.37–1.01)	137	148	149	144	0.98 (0.60–1.61)	145	143	149	141	0.70 (0.42–1.15)
Urban	22	24	25	31	0.53 (0.29–0.96)	26	26	23	27	0.90 (0.50–1.63)	31	24	22	25	0.63 (0.35–1.15)
NSAID use^a^															
None or 1/week	89	84	92	92	1.00 (Reference)	81	96	92	88	1.00 (Reference)	90	93	88	86	1.00 (Reference)
>1/week	111	113	106	104	0.85 (0.66–1.10)	118	104	106	106	0.71 (0.55–0.92)	106	98	116	114	0.77 (0.60–0.99)
Statin use^a^															
None	150	148	149	147	1.00 (Reference)	152	142	142	158	1.00 (Reference)	137	156	152	149	1.00 (Reference)
>1/week	57	52	52	54	0.89 (0.67–1.18)	50	62	62	41	1.38 (1.04–1.83)	63	45	54	53	0.80 (0.60–1.05)

^a^ Sample sizes for some of the covariates are not 813 due to unknown covariate measures.

### 3.4 Multivariable models

**Figure 1:**
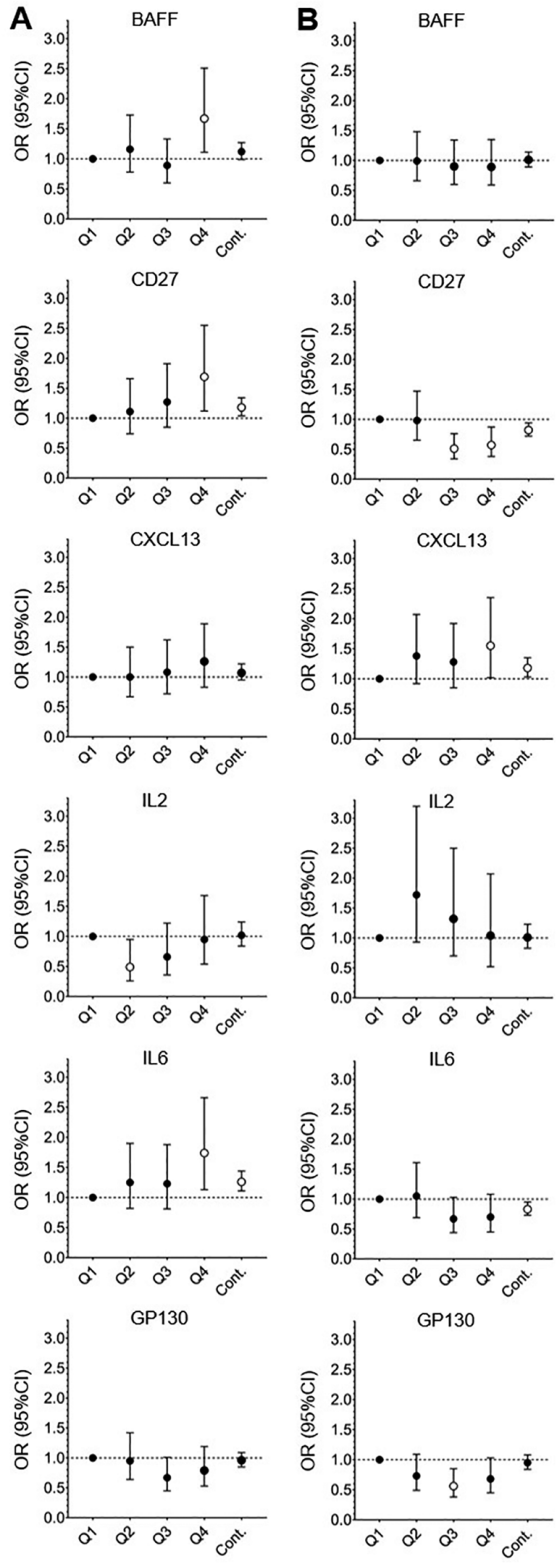
Significant associations from the multivariable association of dichotomized cytokines and log-transformed BFR congeners for the participants in the California Teachers Study who had a serum sample collected from 2013 to 2016. **(A)** Significant quartile 1, 2, 3, 4 (Q1–4, respectively) and continuous model (Cont.) results for BDE47. **(B)** Significant quartile 1, 2, 3, 4 and continuous model results for PBB153. Quartile model: adjusted for age, BMI, and total lipids. Continuous model: BFR congeners = per 1 unit increase (pg/ml), cytokines are dichotomized as above or below the median; adjusted for age and total lipids. BDE47 models were additionally adjusted for PBB153, and PBB153 quartile models were additionally adjusted for BDE47. Open circle symbols indicate a significant association, while closed symbols indicate a non-significant association. Errors bars indicate 95% confidence intervals (95% CI) for the respective odds ratio (OR). Dashed horizontal line indicates OR = 1.00 (null results).

Results of multivariable analyses evaluating the association between BDE153, BDE47 and PBB153 with each immune marker are shown in Supplementary Table S4; results for BDE47 and PBB153 are displayed graphically in Figure 1. Increasing levels of BDE47 were significantly associated with increasing levels of circulating sCD27 (OR_quartile4_ = 1.69, 95% CI = 1.12–2.55), IL6 (OR_quartile4_ = 1.74 95% CI = 1.13–2.66), and BAFF (OR_quartile4_ = 1.67, 95% CI = 1.11–2.51). PBB153 was associated with higher levels of CXCL13 (OR_quartile4_ = 1.18, 95% CI = 1.02–2.35) and lower levels of sCD27 (OR_quartile4_ = 0.57, 95% CI = 0.38–0.87). No significant results were observed for BDE153 and any immune marker (Supplementary Table S4). Continuous models [continuous BFR congeners (pg/ml) and dichotomized immune markers] resulted in relatively similar trends as the logistic models; specifically, in continuous models, for each unit (pg/ml) of increase in BDE47, we observed an odds ratio of 1.26 (95% CI = 1.11–144) and 1.18 (95% CI = 1.04–1.34) for IL6 and sCD27, respectively (Figure 1 and Supplementary Table S4). Multivariable models stratified by key participant characteristics (age, BMI, statin use) are presented in Supplementary Table S6. Overall, the magnitudes of effect were consistent across strata for the indicated immune markers (e.g., BDE47 and sCD27, IL6 and BAFF). We further present the associations between the participant characteristics and the dichotomized immune markers in Supplementary Table S7.

### 3.5 BFR and immune pathways

Elevated levels of BDE47 (quartiles) were associated with the B cell activation pathway (OR_quartile 4_ = 1.59, 95% CI = 1.06–2.39) (Table 3). Consistent with associations with individual cytokines, there were no associations present between BDE153 and any of the immune pathways. We also observed no consistent trends or major associations between PBB153 with any immune pathway.

**Table 3:** Multivariable logistic and continuous models of the association of immune pathways and log-transformed BFR congeners for the participants (*N* = 813) in the California teachers study who had a serum sample, cytokines measured and BFR congeners measured from 2013 to 2016.

BFR congeners	Multivariable immune pathway associations							
	Th1^a^		Pro-inflammatory/macrophage activation^b^		B cell activation^c^		T cell activation^d^	
	OR	95% CI	OR	95% CI	OR	95% CI	OR	95% CI
BDE153								
Quartile 1^e^	1.00	Reference	1.00	Reference	1.00	Reference	1.00	Reference
Quartile 2	1.20	(0.51–2.80)	1.08	(0.72–1.63)	1.01	(0.68–1.51)	0.87	(0.57–1.32)
Quartile 3	1.00	(0.41–2.43)	1.30	(0.86–1.96)	1.11	(0.75–1.66)	0.93	(0.61–1.42)
Quartile 4	1.71	(0.75–3.87)	1.20	(0.79–1.81)	1.13	(0.75–1.69)	0.92	(0.60–1.41)
Continuous^f^	1.19	(0.91–1.56)	1.00	(0.87–1.16)	0.97	(0.85–1.12)	0.92	(0.79–1.06)
BDE47								
Quartile 1	1.00	Reference	1.00	Reference	1.00	Reference	1.00	Reference
Quartile 2	0.74	(0.35–1.57)	0.90	(0.59–1.36)	1.05	(0.71–1.57)	0.83	(0.54–1.27)
Quartile 3	0.21	(0.07–0.64)	0.86	(0.57–1.31)	1.07	(0.71–1.59)	0.79	(0.51–1.21)
Quartile 4	0.88	(0.42–1.85)	1.07	(0.71–1.63)	1.59	(1.06–2.39)	1.23	(0.80–1.87)
Continuous	0.92	(0.71–1.19)	1.09	(0.96–1.24)	1.22	(1.07–1.38)	1.13	(1.00–1.29)
PBB153								
Quartile 1	1.00	Reference	1.00	Reference	1.00	Reference	1.00	Reference
Quartile 2	0.92	(0.42–2.01)	1.26	(0.84–1.90)	1.18	(0.79–1.77)	1.41	(0.93–2.13)
Quartile 3	0.91	(0.41–2.02)	0.91	(0.60–1.38)	0.63	(0.42–0.94)	0.81	(0.53–1.25)
Quartile 4	0.62	(0.25–1.51)	0.82	(0.53–1.25)	0.75	(0.50–1.14)	0.79	(0.51–1.23)
Continuous	0.92	(0.72–1.18)	0.89	(0.78–1.01)	0.95	(0.84–1.08)	0.88	(0.77–1.00)

^a^ Th1: IFN-γ, IL10, and IL4.

^b^ Pro-inflammatory/Macrophage activation: TNF-α, TNF-R2, IL6, IL-1β, IL8, IL6Rα, IL10, and CD14.

^c^ B-cell activation: BAFF, IL10, IL4, IL6, sCD27, CXCL13.

^d^ T-cell activation: IL2, IL2Rα, IFN-γ, IL4, IL6.

^e^ Quartiles: adjusted for age, BMI, and total lipids.

^f^ Continuous: per 1 unit increase (pg/ml), adjusted for age and total lipids.

## 4 Discussion

**Figure 2:**
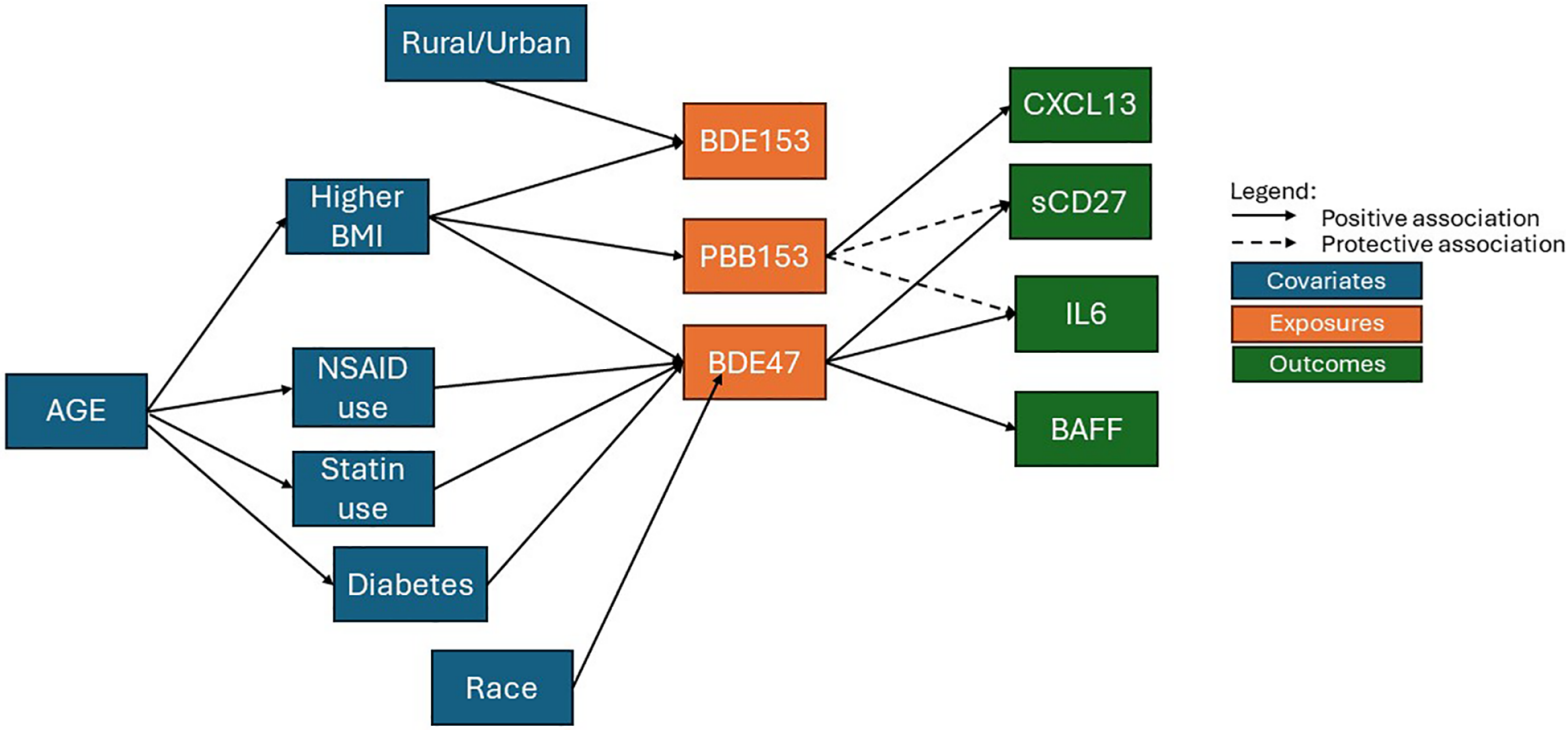
A directed acyclic graph (DAG) representing the complex associations between participant characteristics, immune markers, and BFR congeners.

In our evaluation of 813 participants from the California Teacher Study whose sera was collected between 2013 and 2016 and measured for 11 BFR congeners and 16 circulating immune/inflammation markers, we found: (1) positive associations between BDE47 exposure and elevated levels of sCD27, IL6, and BAFF, (2) inverse associations between PBB153 exposure and sCD27 and IL6 but positive associations with CXCL13, and (3) no associations between BDE153 exposure and any immune marker evaluated. To clarify the complexities of the associations between participant characteristics, immune markers and BFR congeners, a directed acyclic graph (DAG) is presented in Figure 2.

The lipid-adjusted means and medians for BDE153, BDE47 and PBB153 appear relatively similar to prior reports in human population samples; however, we note that corresponding values from our older population of women were higher than national sampling data from the NHANES study in White non-Hispanic women (ages 16–49 years old) (4, 6). Compared to comparable age- and sex-matched data, our population exposure levels were relatively higher than those presented in NHANES data from 2013 to 2015 (Supplementary Table S5). This is expected as California populations have previously been shown to possess higher levels of BFR congeners and PBDE levels when compared to the national averages (4, 12, 33).

Our evaluation of participant characteristics and BFR congener exposure adds substantively to the current literature. Specifically, our results support the positive association observed between non-White races with elevated levels of BDE47 (4, 31, 32). Sjodin et al. (2008) previously reported non-Hispanic Black and Mexican Americans having higher levels of BFR congeners than non-Hispanic Whites, although this may be attributable to oversampling of the NHANES data for these demographic groups (4). The positive association between higher levels of BMI and BDE47 exposure also supports findings previously reported in several studies (31, 32). The association reported in our study of higher levels of BMI and decreased levels of PBB153 have also been previously suggested in a study measuring PBB exposure in 861 chemical workers, farmers and community members (ages 7–88) in Michigan, United States between 2012 and 2015, though these results were not statistically significant (29). Additionally, none of the population characteristics included in our study were associated with the total lipid variable included in the multivariable models. BFR congeners are commonly produced and used within commercial mixtures, thus congeners are typically correlated. Within our data, we report no notable correlations between BDE153, BDE47, and/or PBB153.

Our data illustrate positive associations between increasing BDE47 exposure and elevated circulating levels of IL6, sCD27, and BAFF. As markers of B-cell activation, their elevation with BDE47 exposure support a potential link between BDE47 and health conditions associated with B-cell activation, such as autoimmune conditions (34). Efforts to delineate if exposure to BDE47 that generates an inflammatory environment that provides an environment triggering the onset of autoimmune conditions are warranted.

Our reported association between BDE47 and IL6 supports prior reports from a number of *in vivo* and *in vitro* studies (15–17, 19, 35). In an *in vivo* study where 24 mice were orally exposed to varied levels (0, 1, 10, 100 mg/kg body weight) of BDE47 via gastric infusion, BDE47 exposure increased the gene expression of IL6 measured by quantitative polymerase chain reaction (PCR) (17). An *in vitro* study that utilized human bronchial epithelial cells exposed to 0.01–10 *μ*g/ml of PBDE congeners for 24 h also assessed levels of IL6 via enzyme-linked immunosorbent assay (ELISA) and found that c-pentaBDE and c-octaBDE both increased IL6 levels (19). Notably, one component of c-pentaBDE is BDE47, and c-octaBDE is composed in part by c-pentaBDE. The association between BDE47 and elevated IL6 has also been suggested in one population study of 103 pregnant women in California, United States between 2011 and 2013, though the association was not statistically significant (5). We note inconsistency in the prior literature among the *in vivo* and *in vitro* studies (15, 16, 35) and suggest that differences in methodologies of administering exposure and their measurements may contribute to these inconsistencies. Notably, our population-based study measures circulating immune markers vs. specific target tissues as measured in prior *in vitro* and *in vivo* studies. To our knowledge, associations between BDE47 exposure and increased levels of sCD27 and BAFF, which are both B-cell activation markers, have not been previously reported in epidemiology-based or *in vitro/in vivo* studies and thus require further confirmation in all study types. In analyses of immune pathways, we observed associations between elevated BDE47 levels and the B-cell activation pathway, consistent with the robust associations between BDE47 and individual cytokines in the pathway (i.e., BAFF and sCD27).

Our data also yielded associations between elevated levels of PBB153 with elevated CXCL13, decreased levels of IL6 and decreased levels of sCD27, the latter of which are notably the opposite association observed for BDE47. Additionally, the inverse association is robust in both multivariable models that adjust for BDE47 and in stratified models whereby associations are evaluated by BDE47 strata. To date, the association between higher levels of PBB153 with higher levels of CXCL13 and lower levels of sCD27 have not been previously reported in epidemiology-based studies or *in vivo/in vitro* studies. An association between PBB153 exposure and IL6 levels has only been reported in one *in vitro* study, though Arita et al. reported the opposite (i.e., increased) levels of IL6 when human placenta cells were exposed to 20 uM of PBB153 (15). While we cannot rule out differences in methodologies in the sparse but inconsistent results across study designs, the potential novel associations warrant replication and further investigation.

Participant characteristics were also interrogated as they, too, were associated with immune markers and in some cases, congeners. We note, however, that characteristics such as statin use, BMI, and diabetes were also associated with increasing age. Indeed, the addition of most characteristics beyond age did not affect the resulting risk estimates. Nevertheless, we further conducted stratified analyses and demonstrated that associations between BDE47 and the main immune markers of interest (sCD27, IL6, and BAFF) were consistent by strata where possible. Finally, we note that there is precedence for varying associations observed for each congener. In addition to the congeners not being correlated in our study, and despite their similarities in chemical structure, there is growing data demonstrating adverse health outcomes resulting from different toxicological pathways and mechanisms (e.g., BDE153 has been shown to impact lipid metabolism, and BDE47 has been associated with increased risk for type 2 diabetes and fatty acid alterations in the liver) (7).

Our study is among the only large epidemiologic studies that have investigated the associations between a wide number of BFR congeners and circulating immune marker levels. Notably, our study population reflects a population with a high exposure prevalence, making it an ideal population for investigating associations with BFR congeners. Our study strengths include our large sample size, the use of a large cohort population subset, the high quality of our biospecimens which were all processed within 28 h and exhibited low pre-analytical variability (over 99.5% protocol compliance), and the use of several multiplex immune marker assays. Limitations to our study include the use of an all-women cohort, thus minimizing the generalizability of the study, and the cross-sectional nature of our study which may not fully capture variance in circulating immune markers. Although an all-female cohort may restrict generalizability, there is value in studying these associations between BFR exposure and immune markers in women due to declining immune response with age and due to the overall susceptibility women have for developing autoimmune conditions compared to men (20). The observed risk emphasizes the need to further investigate the immune-related biological pathways that BFR can negatively impact, especially in women. Although BFR congener exposure can also occur via dietary intake (3), and such data would have complemented our analyses, the measurement of BFR in circulating blood does reflect a snapshot of a person's exposure from multiple sources, including diet. Finally, we cannot exclude the possibility that these results may be due to chance; our study integrates multiple comparisons and the hypotheses generated from our data requires replication. In addition to replicating our data, future research should incorporate mechanistic studies to expand the findings reported here. Importantly, future efforts to understand the functional relevance of common combinations of BFRs on immune responses are needed.

## 5 Conclusions

In summary, our data show that measurable BFR congeners, specifically BDE47 and PBB153, are associated with specific circulating immune markers, and that these associations differ by BFR congener. Given their pervasiveness, understanding how BFR exposures and specific congeners may contribute to health endpoints is critical, and our results suggest a role in immune alterations as part of this process. Our results add to prior *in vivo* and *in vitro* studies that have reported potential associations with immune markers with BFR congener exposure; however, expanded human and epidemiologic studies are needed to confirm our novel results, and to compare the biological effects between congeners with relatively similar chemical structures (BDE153 vs. PBB153).

## Data Availability

All data associated with this publication are available for research use. The California Teachers Study welcomes all inquiries (https://www.calteachersstudy.org/for-researchers). Additionally, the PFAS analyte dataset presented in this study can be found in the online Human Health Exposure Analysis Resource data repository (https://hhearprogram.org/data-services). Data associated with this publication are also publicly available through the Human Health Exposure Analysis Resource (HHEAR) Data Center (https://hheardatacenter.mssm.edu/).
