# Supplementary material for: Associations between brominated flame retardants, including polybrominated diphenyl ethers, and immune responses among women in the California Teachers Study
Source: Front Epidemiol. 2025 Mar 19;5:1452934. doi: 10.3389/fepid.2025.1452934 (PMC11962006; doi:10.3389/fepid.2025.1452934)
Supplement: Supplementary file 1 [file Datasheet1.pdf]

Supplementary Tables

Supplementary Table 1. Descriptive statistics (pg/mL) for serum measurements of 11 BFR analytes in a subset of 814 participants from the California Teachers Study from sera collected from 2013-2016.

Analyte	N	N >LOD[a]	Percent Detected	LOD[a]	Min[b]	10th[d]	25th[d]	Median	75th[d]	90th[d]	Max[c]
BDE100	814	349	43%	17	<LOD	<LOD	<LOD	<LOD	29.5	59.1	1250
BDE153	814	639	79%	18	<LOD	<LOD	19.5	33.4	60.5	138	3100
BDE154	814	87	11%	11	<LOD	<LOD	<LOD	<LOD	<LOD	11.7	302
BDE17	814	26	3%	12	<LOD	<LOD	<LOD	<LOD	<LOD	<LOD	131
BDE183	814	41	5%	14	<LOD	<LOD	<LOD	<LOD	<LOD	<LOD	324
BDE28	814	182	22%	13	<LOD	<LOD	<LOD	<LOD	<LOD	20.7	182
BDE47	814	668	82%	25	<LOD	<LOD	32.8	70.3	137	263	6130
BDE66	814	25	3%	19	<LOD	<LOD	<LOD	<LOD	<LOD	<LOD	189
BDE85	814	74	9%	16	<LOD	<LOD	<LOD	<LOD	<LOD	<LOD	191
BDE99	814	330	41%	17	<LOD	<LOD	<LOD	<LOD	27.5	62.6	1680
PBB153	814	602	74%	12	<LOD	<LOD	<LOD	18.6	26.9	41.8	5410

[a]LOD: Limit of detection. [b]Min: Minimum value. [c]Max: Maximum value. [d]10th, 25th, 75th and 90th: respective percentiles.
Analyte legend: BFR: brominated flame retardants, BDE100: 2,2',4,4',6-Pentabromodiphenyl ether, BDE153: 2,2',4,4',5,5'-Hexabromodiphenyl ether, BDE154: 2,2',4,4',5,6'-Hexabromodiphenyl ether, BDE17: 2,2',4-Tribromodiphenyl ether, BDE183: 2,2',3,4,4',5',6-Heptabromodiphenyl ether, BDE28: 2,4,4'-Tribromodiphenyl ether, BDE47: 2,2',4,4'-Tetrabromodiphenyl ether, BDE66: 2,3',4,4'-Tetrabromodiphenyl ether, BDE85: 2,2',3,4,4'-Pentabromodiphenyl ether, BDE99: 2,2',4,4',5-Pentabromodiphenyl ether, and PBB153: 2,2',4,4',5,5'-Hexabromobiphenyl

Supplementary Tables

Supplementary Table 2. Descriptive statistics (pg/mL) for 16 serum-based cytokines and immune markers measured in a subset of 813 participants[a] in the California Teachers Study cohort who had serum collected from 2013-2016.

Label	N	Min	10th	25th	Median	75th	90th	Max
**BAFF**	813	401.34	1101.51	1260.33	1471.72	1720.13	1959.23	3205.82
**TNF-R2**	813	0	1945.9	2334.5	2782.99	3313	4044.15	27857.45
**TNF-α**	813	0.1	5.25	6.78	8.41	10.51	13.07	51.31
**sCD14**[b]	812	26236.13	791122.3	913659.91	1053100	1186100	1309600	2399000
**sCD27**	813	1607.92	5456.6	6487.33	7793.23	9580	11753.99	32333.37
**CXCL13**	813	0	18.95	31.75	43.59	58.6	75.35	666.95
**IFN-γ**	813	0	0	0	0	0	0	226.845
**IL2**	813	0	0	0	0	0.61	1.07	20.33
**IL4**	813	0	0	0	0	0	0	50.3
**IL6**	813	0	0.73	1.17	1.74	2.54	3.65	402.05
**IL8**	813	2.21	6.19	8.17	11.18	15.94	22.5	5619.54
**IL10**	813	0	0.1	0.21	0.32	0.49	0.73	365.94
**IL-1β**	813	0	0.23	0.34	0.54	0.86	1.54	37
**IL2Rα**	813	267.68	715.25	854.09	1092.8	1393.82	1753.54	3856
**IL6Rα**	813	3489.36	33961.73	40248.08	48903.88	58672.95	68738.22	138716.09
**GP130**	813	24090.24	184801.6	210041.56	234157	260190.3	286650.5	484779.7

[a]814 participants were included in the BFR analytes measurement, but only 813 participants were included in the cytokine measurements. [b]CD14: N=812, 1 participant was dropped due to missing cytokine measurement.

Supplementary Table 3. Spearman's correlation coefficient between each BFR analyte from levels ascertained from participants (N=813) in the California Teachers study who had a serum sample collected from 2013-2016.

	BDE100	BDE153	BDE154	BDE17	BDE183	BDE28	BDE47	BDE66	BDE85	BDE99	PBB153
BDE100	1.00	0.63	0.62	0.56	0.31	0.85	0.88	0.43	0.63	0.91	0.10
BDE153		1.00	0.40	0.30	0.09	0.45	0.48	0.23	0.44	0.60	0.23
BDE154			1.00	0.34	0.31	0.50	0.51	0.22	0.68	0.62	0.27
BDE17				1.00	0.23	0.62	0.43	0.55	0.28	0.54	-0.03
BDE183					1.00	0.23	0.16	0.31	0.36	0.30	0.13
BDE28						1.00	0.89	0.38	0.53	0.79	0.06
BDE47							1.00	0.25	0.57	0.85	0.06
BDE66								1.00	0.25	0.39	-0.07
BDE85									1.00	0.69	0.32
BDE99										1.00	0.17
PBB153											1.00

Supplementary Table 4. Multivariable association of log-transformed BFR analytes (exposure expressed as quartiles) and immune markers (outcomes are dichotomized) among blood from participants (N=813) in the California Teachers Study (CTS) who participated in the CTS biobanking study by providing blood, from 2013-2016.

Multivariable Cytokine-BFR Associations																
	**BAFF**		**TNF-R2**		**TNF-α**		**CD14[a]**		**CD27**		**CXCL13**		**IFN-γ**		**IL2**	
	**OR**	**95% CI**	**OR**	**95% CI**	**OR**	**95% CI**	**OR**	**95% CI**	**OR**	**95% CI**	**OR**	**95% CI**	**OR**	**95% CI**	**OR**	**95% CI**
BDE153																
Quartile 1[b]	1.00	Reference	1.00	Reference	1.00	Reference	1.00	Reference	1.00	Reference	1.00	Reference	1.00	Reference	1.00	Reference
Quartile 2	1.00	(0.67-1.49)	1.04	(0.69-1.58)	0.86	(0.58-1.29)	1.16	(0.78-1.72)	0.91	(0.61-1.35)	0.97	(0.65-1.45)	1.32	(0.50-3.44)	0.94	(0.50-1.76)
Quartile 3	1.20	(0.81-1.79)	1.12	(0.74-1.71)	1.08	(0.72-1.61)	0.95	(0.64-1.42)	0.75	(0.51-1.13)	1.14	(0.76-1.71)	0.94	(0.33-2.67)	1.31	(0.72-2.38)
Quartile 4	1.28	(0.86-1.91)	1.48	(0.97-2.25)	0.98	(0.66-1.47)	0.84	(0.56-1.26)	1.11	(0.74-1.67)	0.88	(0.59-1.32)	1.72	(0.67-4.42)	0.88	(0.47-1.68)
Continuous[c]	1.04	(0.90-1.19)	1.06	(0.92-1.22)	0.98	(0.85-1.13)	0.96	(0.84-1.11)	1.02	(0.89-1.17)	1.00	(0.87-1.15)	1.28	(0.94-1.74)	1.01	(0.81-1.25)
BDE47[d]																
Quartile 1	1.00	Reference	1.00	Reference	1.00	Reference	1.00	Reference	1.00	Reference	1.00	Reference	1.00	Reference	1.00	Reference
Quartile 2	1.16	(0.78-1.73)	0.83	(0.55-1.27)	0.77	(0.52-1.15)	0.91	(0.61-1.35)	1.11	(0.74-1.66)	1.00	(0.67-1.50)	0.94	(0.37-2.38)	0.49	(0.26-0.95)
Quartile 3	0.89	(0.60-1.33)	1.07	(0.70-1.63)	0.86	(0.58-1.29)	1.00	(0.67-1.48)	1.27	(0.85-1.91)	1.08	(0.72-1.62)	0.64	(0.22-1.81)	0.66	(0.36-1.22)
Quartile 4	1.67	(1.11-2.51)	1.02	(0.66-1.55)	0.92	(0.61-1.39)	0.86	(0.57-1.28)	1.69	(1.12-2.55)	1.26	(0.83-1.89)	1.21	(0.50-2.95)	0.95	(0.54-1.68)
Continuous	1.12	(0.99-1.27)	1.10	(0.97-1.25)	1.00	(0.88-1.13)	0.96	(0.84-1.08)	1.18	(1.04-1.34)	1.07	(0.95-1.22)	1.11	(0.82-1.50)	1.02	(0.84-1.24)
PBB153[e]																
Quartile 1	1.00	Reference	1.00	Reference	1.00	Reference	1.00	Reference	1.00	Reference	1.00	Reference	1.00	Reference	1.00	Reference
Quartile 2	0.99	(0.66-1.48)	1.28	(0.84-1.94)	1.23	(0.82-1.84)	0.73	(0.49-1.09)	0.98	(0.65-1.47)	1.38	(0.92-2.07)	0.92	(0.36-2.34)	1.72	(0.93-3.20)
Quartile 3	0.90	(0.60-1.34)	0.75	(0.49-1.15)	1.13	(0.76-1.70)	0.79	(0.53-1.18)	0.51	(0.34-0.76)	1.28	(0.85-1.92)	1.19	(0.49-2.90)	1.32	(0.70-2.50)
Quartile 4	0.89	(0.59-1.35)	1.10	(0.71-1.69)	1.09	(0.72-1.64)	0.84	(0.56-1.27)	0.57	(0.38-0.87)	1.55	(1.02-2.35)	0.66	(0.22-1.93)	1.04	(0.52-2.07)
Continuous	1.01	(0.89-1.14)	0.94	(0.82-1.07)	0.93	(0.82-1.06)	0.94	(0.83-1.07)	0.82	(0.72-0.94)	1.18	(1.03-1.35)	0.95	(0.72-1.26)	1.01	(0.83-1.23)
	**IL4**		**IL6**		**IL8**		**IL10**		**IL-1β**		**IL2Rα**		**IL6Rα**		**GP130**	
	**OR**	**95% CI**	**OR**	**95% CI**	**OR**	**95% CI**	**OR**	**95% CI**	**OR**	**95% CI**	**OR**	**95% CI**	**OR**	**95% CI**	**OR**	**95% CI**
BDE153																
Quartile 1	1.00	Reference	1.00	Reference	1.00	Reference	1.00	Reference	1.00	Reference	1.00	Reference	1.00	Reference	1.00	Reference
Quartile 2	0.98	(0.42-2.31)	1.49	(0.98-2.27)	0.92	(0.62-1.38)	0.79	(0.53-1.17)	0.92	(0.62-1.37)	0.81	(0.54-1.21)	0.99	(0.67-1.47)	0.72	(0.48-1.07)
Quartile 3	1.32	(0.59-2.94)	1.40	(0.92-2.14)	0.90	(0.60-1.35)	0.94	(0.64-1.40)	1.04	(0.70-1.55)	0.77	(0.52-1.16)	0.95	(0.64-1.42)	0.93	(0.62-1.38)
Quartile 4	0.97	(0.42-2.25)	1.12	(0.73-1.70)	1.08	(0.72-1.62)	0.88	(0.59-1.31)	0.86	(0.57-1.28)	1.10	(0.74-1.66)	0.88	(0.59-1.31)	0.93	(0.62-1.39)
Continuous	0.93	(0.69-1.25)	0.91	(0.79-1.04)	1.05	(0.91-1.21)	1.02	(0.88-1.17)	0.93	(0.81-1.07)	1.01	(0.88-1.16)	0.96	(0.83-1.10)	1.07	(0.93-1.23)
BDE47																
Quartile 1	1.00	Reference	1.00	Reference	1.00	Reference	1.00	Reference	1.00	Reference	1.00	Reference	1.00	Reference	1.00	Reference
Quartile 2	0.52	(0.23-1.18)	1.25	(0.82-1.90)	0.73	(0.49-1.09)	0.95	(0.64-1.41)	0.75	(0.50-1.12)	0.91	(0.60-1.36)	1.00	(0.67-1.49)	0.95	(0.64-1.42)
Quartile 3	0.48	(0.21-1.11)	1.23	(0.81-1.88)	0.71	(0.47-1.07)	0.74	(0.50-1.10)	0.91	(0.61-1.36)	0.93	(0.62-1.40)	0.96	(0.65-1.44)	0.67	(0.45-1.01)
Quartile 4	0.64	(0.29-1.42)	1.74	(1.13-2.66)	0.87	(0.58-1.32)	0.82	(0.55-1.22)	1.09	(0.73-1.64)	1.08	(0.72-1.62)	0.91	(0.61-1.37)	0.79	(0.53-1.19)
Continuous	0.90	(0.69-1.16)	1.26	(1.11-1.44)	0.98	(0.86-1.11)	1.00	(0.89-1.14)	1.04	(0.92-1.18)	1.04	(0.92-1.18)	1.00	(0.88-1.13)	0.96	(0.85-1.09)
PBB153																
Quartile 1	1.00	Reference	1.00	Reference	1.00	Reference	1.00	Reference	1.00	Reference	1.00	Reference	1.00	Reference	1.00	Reference
Quartile 2	1.40	(0.66-3.00)	1.05	(0.69-1.61)	0.97	(0.65-1.46)	1.25	(0.84-1.86)	1.33	(0.89-1.98)	1.41	(0.94-2.12)	1.24	(0.83-1.85)	0.73	(0.49-1.09)
Quartile 3	0.64	(0.26-1.57)	0.67	(0.44-1.03)	0.93	(0.62-1.40)	0.84	(0.56-1.25)	1.02	(0.68-1.52)	0.90	(0.60-1.35)	0.81	(0.54-1.21)	0.56	(0.38-0.85)
Quartile 4	0.67	(0.28-1.64)	0.70	(0.45-1.08)	1.27	(0.84-1.94)	1.05	(0.70-1.58)	1.25	(0.83-1.88)	1.14	(0.75-1.73)	0.73	(0.48-1.11)	0.68	(0.45-1.03)
Continuous	0.88	(0.69-1.14)	0.83	(0.73-0.95)	1.10	(0.96-1.26)	1.01	(0.89-1.15)	0.99	(0.88-1.13)	1.00	(0.88-1.14)	0.91	(0.80-1.04)	0.95	(0.84-1.08)

[a]CD14: N=812, 1 participant was dropped due to missing cytokine measurement. [b]Quartiles: adjusted for age, BMI, and total lipids. [c]Continuous: per 1 unit increase (pg/mL), adjusted for age and total lipids. [d]BDE47 quartile and continuous models were additionally adjusted for PBB153. [e]PBB153 quartile and continuous models were additionally adjusted for BDE47.

Supplementary Table 5. Descriptive statistics for age-and sex-matched serum measurements of BFR analytes across this study (N=814, pg/mL), NHANES 2013 data (N=83, pg/g), and NHANES 2015 data (N=79, pg/g).

Analyte	Minimum	10th[a]	25th[a]	Median	75th[a]	90th[a]	Maximum
Current study – 2013-2016							
BDE153	<LOD	<LOD	19.6	33.4	60.5	138	3100
BDE47	<LOD	<LOD	32.8	70.4	137	263	6130
PBB153	<LOD	<LOD	<LOD	18.6	26.7	41.8	5410
NHANES - 2013[b]							
BDE153	14.9	26.9	37.2	59.7	80.6	126.5	200.7
BDE47	26.0	48.0	71.8	94.6	157.7	198.6	862.8
PBB153	3.5	9.6	12.8	18.7	43.2	77.3	246.4
NHANES - 2015[b]							
BDE153	11.4	27.9	36.7	61.3	101.9	146.5	234.5
BDE47	18.1	39.9	59.0	85.8	135.7	241.3	679.9
PBB153	2.9	8.6	11.2	14.6	22.5	28.2	139.3

[a]10th, 25th, 75th and 90th: respective percentiles.

[b]NHANES data consisted of pooled samples, restricted to an age- and sex-matched population to our study; descriptive statistics were calculated using respective survey weights; data can be found at https://wwwn.cdc.gov/Nchs/Data/Nhanes/Public/2013/DataFiles/BFRPOL_H.htm and https://wwwn.cdc.gov/Nchs/Data/Nhanes/Public/2015/DataFiles/BFRPOL_I.htm

Analyte legend: BFR: brominated flame retardants, BDE153: 2,2',4,4',5,5'-Hexabromodiphenyl ether, BDE47: 2,2',4,4'-Tetrabromodiphenyl ether, BDE66: 2,3',4,4'-Tetrabromodiphenyl ether, and PBB153: 2,2',4,4',5,5'-Hexabromobiphenyl

Supplemental Table 6. Multivariable associations between BDE47 (quartile exposure) and immune markers (dichotomized above/below respective median) stratified by key participant covariates.

		Statin (no)			Statin (yes)			BMI (<25 kg/m^2)			BMI (≥25 kg/m^2)			Age (<61 years old)			Age (≥ 61 years old)		
		**OR**	**L**	**U**	**OR**	**L**	**U**	**OR**	**L**	**U**	**OR**	**L**	**U**	**OR**	**L**	**U**	**OR**	**L**	**U**
**Baff**	**Q2**	1.10	0.70	1.73	1.32	0.55	3.14	1.06	0.62	1.81	1.28	0.70	2.34	1.11	0.61	2.01	1.25	0.72	2.19
	**Q3**	0.79	0.49	1.28	1.11	0.49	2.51	0.75	0.42	1.33	0.99	0.56	1.74	1.15	0.61	2.14	0.72	0.42	1.22
	**Q4**	1.62	1.01	2.62	1.59	0.68	3.71	1.93	1.04	3.61	1.58	0.90	2.80	1.81	0.99	3.32	1.58	0.89	2.78
**TNF-R2**	**Q2**	0.71	0.44	1.15	1.52	0.59	3.91	0.85	0.48	1.51	0.76	0.40	1.45	1.15	0.60	2.19	0.67	0.38	1.20
	**Q3**	1.02	0.63	1.67	1.35	0.56	3.25	1.32	0.73	2.40	0.86	0.47	1.57	1.99	1.03	3.86	0.67	0.39	1.17
	**Q4**	0.91	0.56	1.50	1.39	0.57	3.40	1.26	0.67	2.40	0.76	0.42	1.39	1.22	0.64	2.35	0.88	0.49	1.58
**TNF-α**	**Q2**	0.70	0.45	1.11	1.02	0.43	2.46	0.70	0.41	1.22	0.87	0.47	1.60	0.77	0.43	1.40	0.81	0.47	1.41
	**Q3**	0.77	0.48	1.23	1.14	0.49	2.62	0.71	0.40	1.27	1.01	0.56	1.81	1.17	0.63	2.17	0.68	0.40	1.16
	**Q4**	0.81	0.51	1.31	1.19	0.51	2.79	1.07	0.58	1.99	0.90	0.51	1.61	1.16	0.64	2.11	0.75	0.43	1.31
**CD14**	**Q2**	0.81	0.52	1.28	1.14	0.48	2.71	0.73	0.43	1.26	1.02	0.56	1.86	1.37	0.76	2.45	0.63	0.36	1.10
	**Q3**	0.88	0.55	1.40	1.37	0.61	3.10	1.17	0.65	2.09	0.86	0.48	1.51	1.17	0.63	2.16	0.88	0.52	1.50
	**Q4**	0.74	0.46	1.19	1.19	0.51	2.76	0.89	0.48	1.64	0.76	0.43	1.34	0.94	0.52	1.72	0.80	0.46	1.40
**CD27**	**Q2**	0.95	0.60	1.51	1.81	0.76	4.34	0.89	0.51	1.56	1.32	0.71	2.44	0.97	0.52	1.82	1.30	0.74	2.26
	**Q3**	1.30	0.81	2.10	1.35	0.60	3.06	1.58	0.87	2.84	0.99	0.56	1.77	1.90	1.00	3.61	0.93	0.55	1.58
	**Q4**	1.62	1.00	2.63	1.88	0.81	4.36	1.81	0.96	3.38	1.58	0.88	2.82	2.30	1.23	4.31	1.23	0.70	2.16
**CXCL13**	**Q2**	1.07	0.67	1.71	0.82	0.34	1.98	1.06	0.61	1.85	1.02	0.55	1.89	0.83	0.46	1.49	1.07	0.62	1.86
	**Q3**	1.16	0.72	1.87	0.78	0.34	1.77	1.41	0.78	2.54	0.93	0.52	1.65	1.07	0.58	1.99	1.05	0.62	1.78
	**Q4**	1.44	0.88	2.34	0.87	0.38	2.02	1.22	0.66	2.27	1.20	0.67	2.14	1.43	0.78	2.64	1.07	0.61	1.86
**IL6**	**Q2**	0.99	0.62	1.60	3.56	1.34	9.47	0.98	0.56	1.72	2.02	1.09	3.76	1.06	0.55	2.02	1.67	0.94	2.97
	**Q3**	1.15	0.71	1.88	1.92	0.80	4.63	0.80	0.44	1.48	2.14	1.19	3.84	1.59	0.81	3.10	1.12	0.65	1.95
	**Q4**	1.62	0.99	2.67	2.64	1.06	6.58	1.20	0.63	2.26	2.80	1.55	5.08	2.73	1.42	5.24	1.22	0.68	2.18
**IL8**	**Q2**	0.75	0.47	1.20	0.46	0.17	1.21	0.88	0.50	1.52	0.59	0.31	1.11	0.99	0.55	1.79	0.62	0.35	1.08
	**Q3**	0.68	0.42	1.10	0.43	0.17	1.08	1.07	0.60	1.93	0.54	0.29	0.98	1.14	0.62	2.12	0.54	0.32	0.93
	**Q4**	1.13	0.70	1.83	0.32	0.13	0.81	0.93	0.50	1.73	0.82	0.45	1.50	0.92	0.50	1.68	0.82	0.47	1.46
**IL10**	**Q2**	0.87	0.56	1.37	1.48	0.63	3.51	0.76	0.44	1.30	1.23	0.67	2.24	0.64	0.36	1.14	1.36	0.78	2.35
	**Q3**	0.59	0.37	0.95	1.46	0.65	3.30	0.65	0.36	1.16	0.88	0.50	1.55	0.60	0.33	1.11	0.87	0.51	1.46
	**Q4**	0.68	0.42	1.09	1.53	0.66	3.54	0.70	0.38	1.28	0.98	0.56	1.72	0.65	0.36	1.17	0.96	0.56	1.67
**IL-1β**	**Q2**	0.71	0.45	1.12	0.87	0.37	2.05	0.87	0.50	1.49	0.63	0.34	1.16	0.66	0.36	1.19	0.76	0.44	1.31
	**Q3**	0.88	0.55	1.41	0.95	0.42	2.13	1.24	0.70	2.21	0.70	0.40	1.24	1.01	0.54	1.88	0.84	0.50	1.43
	**Q4**	1.11	0.69	1.77	0.91	0.40	2.10	1.03	0.56	1.89	0.97	0.55	1.72	0.82	0.45	1.51	1.35	0.78	2.34
**IL2Rα**	**Q2**	0.95	0.60	1.51	0.80	0.32	1.97	0.91	0.53	1.58	0.86	0.47	1.61	1.41	0.76	2.63	0.69	0.39	1.23
	**Q3**	0.99	0.62	1.59	0.80	0.34	1.87	1.25	0.70	2.24	0.68	0.38	1.21	2.46	1.29	4.69	0.45	0.26	0.79
	**Q4**	1.04	0.64	1.67	0.97	0.41	2.31	1.41	0.76	2.61	0.74	0.42	1.32	2.25	1.20	4.23	0.60	0.34	1.07
**IL6Rα**	**Q2**	1.02	0.65	1.60	1.05	0.44	2.53	1.01	0.59	1.74	1.03	0.56	1.91	1.04	0.58	1.86	1.02	0.59	1.77
	**Q3**	1.11	0.70	1.78	0.78	0.34	1.78	0.75	0.42	1.34	1.26	0.70	2.26	0.98	0.53	1.81	0.94	0.56	1.60
	**Q4**	0.93	0.58	1.49	0.86	0.37	2.00	0.91	0.49	1.68	0.96	0.54	1.71	0.94	0.52	1.71	0.90	0.52	1.57
**GP130**	**Q2**	1.02	0.64	1.61	0.67	0.27	1.65	1.16	0.67	2.00	0.74	0.40	1.37	0.86	0.48	1.55	1.14	0.65	2.00
	**Q3**	0.81	0.50	1.30	0.33	0.14	0.78	0.66	0.37	1.18	0.71	0.40	1.27	0.79	0.43	1.46	0.63	0.37	1.08
	**Q4**	0.76	0.47	1.24	0.60	0.25	1.43	0.65	0.35	1.20	0.86	0.48	1.54	0.74	0.41	1.35	0.83	0.47	1.45

Q2/Q3/Q4= BDE47 quartile; Q1 is the reference (OR=1.00). L= lower limit of the 95% CI; U= upper limit of the 95% CI. IFN-γ, IL2, and IL4 associations could not be stratified due to insufficient numbers. Diabetes status stratification could not be completed due to insufficient numbers.

Supplemental Table 7. Univariate associations between participant characteristics and immune markers (dichotomized above/below the median) measured in the serum of 813* women in the California Teachers Study.

	**Baff**<br>**OR (95% CI)**	**TNF-R2**<br>**OR (95% CI)**	**TNF-α**<br>**OR (95% CI)**	**CD14**<br>**OR (95% CI)**	**CD27**<br>**OR (95% CI)**	**CXCL13**<br>**OR (95% CI)**	**IFN-γ**<br>**OR (95% CI)**	**IL2**<br>**OR (95% CI)**
**Race**								
Non-Hispanic White	1.00 (Reference)	1.00 (Reference)	1.00 (Reference)	1.00 (Reference)	1.00 (Reference)	1.00 (Reference)	1.00 (Reference)	1.00 (Reference)
Other	1.11(0.79-1.55)	0.64(0.45-0.90)	1.03(0.73-1.45)	0.68(0.49-0.96)	0.72(0.51-1.01)	0.85(0.61-1.2)	0.73(0.30-1.78)	0.81(0.47-1.41)
**Age (years)**								
40-49	1.00 (Reference)	1.00 (Reference)	1.00 (Reference)	1.00 (Reference)	1.00 (Reference)	1.00 (Reference)	1.00 (Reference)	1.00 (Reference)
50-59	1.12(0.70-1.79)	1.64(0.99-2.72)	1.23(0.77-1.98)	1.26(0.79-2.02)	1.22(0.75-1.96)	0.42(0.26-0.69)	0.37(0.14-0.98)	0.66(0.35-1.26)
60-69	1.42(0.92-2.22)	3.17(1.97-5.12)	1.51(0.97-2.35)	1.45(0.93-2.25)	1.89(1.20-2.95)	0.40(0.25-0.63)	0.51(0.22-1.17)	0.51(0.28-0.95)
70+	1.88(1.09-3.23)	5.92(3.29-10.67)	2.45(1.42-4.24)	1.41(0.83-2.42)	2.48(1.43-4.29)	0.43(0.24-0.74)	0.28(0.07-1.06)	0.55(0.25-1.20)
**Socioeconomic status (SES)***								
Quartile 1	1.00 (Reference)	1.00 (Reference)	1.00 (Reference)	1.00 (Reference)	1.00 (Reference)	1.00 (Reference)	1.00 (Reference)	1.00 (Reference)
Quartile 2	0.24(0.09-0.60)	0.80(0.35-1.85)	0.66(0.28-1.55)	0.38(0.16-0.89)	0.73(0.32-1.67)	0.50(0.21-1.18)	0.72(0.07-7.16)	0.89(0.27-2.93)
Quartile 3	0.37(0.15-0.91)	0.71(0.33-1.56)	0.56(0.25-1.26)	0.60(0.27-1.33)	0.80(0.37-1.76)	0.65(0.29-1.46)	1.58(0.20-12.45)	0.72(0.23-2.22)
Quartile 4	0.32(0.13-0.77)	0.72(0.33-1.57)	0.52(0.23-1.16)	0.77(0.35-1.70)	0.68(0.31-1.48)	0.49(0.22-1.09)	1.23(0.16-9.57)	0.80(0.27-2.41)
**Diabetes***								
No	1.00 (Reference)	1.00 (Reference)	1.00 (Reference)	1.00 (Reference)	1.00 (Reference)	1.00 (Reference)	1.00 (Reference)	1.00 (Reference)
Yes	2.35(1.42-3.9)	3.36(1.96-5.77)	3.27(1.91-5.61)	0.79(0.49-1.28)	3.14(1.85-5.34)	1.2(0.75-1.92)	1.55(0.58-4.09)	1.47(0.76-2.84)
**Body mass index (BMI, kg/m^2)***								
15-24	1.00 (Reference)	1.00 (Reference)	1.00 (Reference)	1.00 (Reference)	1.00 (Reference)	1.00 (Reference)	1.00 (Reference)	1.00 (Reference)
25-29	0.76(0.55-1.06)	1.38(1.00-1.92)	1.63(1.17-2.25)	0.83(0.60-1.15)	1.36(0.98-1.88)	1.74(1.26-2.42)	0.84(0.37-1.90)	1.41(0.86-2.30)
30+	1.60(1.11-2.3)	3.32(2.26-4.87)	1.76(1.23-2.54)	0.52(0.36-0.74)	1.87(1.30-2.70)	1.52(1.06-2.18)	1.16(0.51-2.65)	0.80(0.43-1.49)
**Physical activity (hr/week)***								
0-2.37	1.00 (Reference)	1.00 (Reference)	1.00 (Reference)	1.00 (Reference)	1.00 (Reference)	1.00 (Reference)	1.00 (Reference)	1.00 (Reference)
2.38-5.88	0.75(0.53-1.05)	0.54(0.38-0.76)	0.66(0.47-0.92)	1.20(0.85-1.68)	0.82(0.58-1.15)	0.59(0.42-0.83)	0.69(0.33-1.46)	0.88(0.52-1.50)
5.88+	0.79(0.56-1.10)	0.63(0.45-0.89)	0.56(0.40-0.79)	1.54(1.10-2.16)	0.62(0.44-0.87)	0.52(0.37-0.73)	0.39(0.16-0.95)	1.08(0.65-1.81)
**Rural/Urban residence***								
Rural	1.00 (Reference)	1.00 (Reference)	1.00 (Reference)	1.00 (Reference)	1.00 (Reference)	1.00 (Reference)	1.00 (Reference)	1.00 (Reference)
Town	1.01(0.28-3.57)	1.93(0.52-7.16)	1.67(0.45-6.19)	1.67(0.45-6.19)	2.23(0.60-8.28)	0.67(0.19-2.34)	N/A	0.54(0.06-4.73)
City	0.85(0.41-1.79)	0.93(0.45-1.94)	0.75(0.36-1.57)	0.66(0.31-1.38)	1.01(0.48-2.10)	0.53(0.25-1.11)	0.61(0.10-3.79)	0.79(0.27-2.35)
Suburban	0.69(0.40-1.22)	0.99(0.57-1.72)	0.84(0.48-1.46)	0.87(0.50-1.52)	1.04(0.60-1.81)	0.70(0.40-1.23)	0.78(0.23-2.68)	0.76(0.34-1.67)
Urban	0.64(0.33-1.24)	0.68(0.35-1.31)	0.80(0.42-1.55)	0.63(0.33-1.22)	1.47(0.76-2.84)	0.45(0.23-0.88)	1.08(0.26-4.51)	0.78(0.30-2.05)
**NSAID use***								
None or 1/week	1.00 (Reference)	1.00 (Reference)	1.00 (Reference)	1.00 (Reference)	1.00 (Reference)	1.00 (Reference)	1.00 (Reference)	1.00 (Reference)
>1/week	1.49(1.12-1.97)	1.26(0.96-1.67)	1.45(1.09-1.92)	1.10(0.83-1.45)	0.94(0.71-1.24)	1.01(0.76-1.33)	0.65(0.33-1.26)	1.13(0.73-1.76)
**Statin use***								
None	1.00 (Reference)	1.00 (Reference)	1.00 (Reference)	1.00 (Reference)	1.00 (Reference)	1.00 (Reference)	1.00 (Reference)	1.00 (Reference)
>1/week	1.44(1.05-1.97)	1.81(1.32-2.49)	2.00(1.45-2.75)	0.89(0.65-1.21)	1.32(0.96-1.80)	1.19(0.87-1.63)	1.40(0.69-2.86)	1.32(0.83-2.10)

HHEAR BFR TABLES

	IL4 OR (95% CI)	IL6 OR (95% CI)	IL8 OR (95% CI)	IL10 OR (95% CI)	IL-1β OR (95% CI)	IL2Rα OR (95% CI)	IL6Rα OR (95% CI)	GP130 OR (95% CI)
**Race**								
Non-Hispanic White	1.00 (Reference)	1.00 (Reference)	1.00 (Reference)	1.00 (Reference)	1.00 (Reference)	1.00 (Reference)	1.00 (Reference)	1.00 (Reference)
Other	0.6(0.27-1.37)	0.98(0.70-1.38)	0.82(0.59-1.15)	0.79(0.56-1.11)	1.23(0.88-1.73)	0.45(0.32-0.65)	0.73(0.52-1.02)	0.61(0.43-0.86)
**Age (years)**								
40-49	1.00 (Reference)	1.00 (Reference)	1.00 (Reference)	1.00 (Reference)	1.00 (Reference)	1.00 (Reference)	1.00 (Reference)	1.00 (Reference)
50-59	1.95(0.54-7)	1.10(0.69-1.76)	2.02(1.21-3.36)	1.06(0.66-1.69)	0.72(0.45-1.15)	1.02(0.64-1.63)	1.26(0.78-2.01)	2.18(1.33-3.59)
60-69	2.86(0.85-9.57)	1.46(0.94-2.27)	3.66(2.25-5.96)	1.35(0.87-2.10)	0.81(0.52-1.26)	1.37(0.88-2.13)	1.48(0.95-2.30)	2.86(1.78-4.60)
70+	1.51(0.35-6.5)	1.81(1.06-3.11)	5.34(2.97-9.60)	1.68(0.98-2.88)	0.66(0.39-1.13)	2.02(1.17-3.47)	2.36(1.37-4.08)	3.55(2.01-6.25)
**Socioeconomic status (SES)***								
Quartile 1	1.00 (Reference)	1.00 (Reference)	1.00 (Reference)	1.00 (Reference)	1.00 (Reference)	1.00 (Reference)	1.00 (Reference)	1.00 (Reference)
Quartile 2	N/A	0.39(0.16-0.95)	0.84(0.37-1.92)	1.00(0.44-2.28)	1.37(0.60-3.14)	1.00(0.44-2.28)	0.51(0.22-1.18)	0.31(0.13-0.77)
Quartile 3	N/A	0.39(0.16-0.91)	0.99(0.46-2.16)	1.04(0.48-2.26)	1.23(0.57-2.69)	0.93(0.43-2.02)	0.70(0.32-1.55)	0.42(0.18-0.99)
Quartile 4	N/A	0.38(0.16-0.89)	1.03(0.48-2.21)	0.95(0.44-2.04)	1.03(0.48-2.21)	0.78(0.36-1.68)	0.64(0.29-1.41)	0.39(0.17-0.91)
**Diabetes***								
No	1.00 (Reference)	1.00 (Reference)	1.00 (Reference)	1.00 (Reference)	1.00 (Reference)	1.00 (Reference)	1.00 (Reference)	1.00 (Reference)
Yes	1.67(0.72-3.86)	3.09(1.82-5.25)	1.92(1.17-3.13)	1.21(0.75-1.94)	1.73(1.07-2.81)	1.68(1.04-2.73)	1.42(0.88-2.29)	1.61(0.99-2.61)
**Body mass index (BMI, kg/m^2)***								
15-24	1.00 (Reference)	1.00 (Reference)	1.00 (Reference)	1.00 (Reference)	1.00 (Reference)	1.00 (Reference)	1.00 (Reference)	1.00 (Reference)
25-29	0.68(0.32-1.45)	1.68(1.21-2.34)	1.23(0.89-1.70)	1.24(0.89-1.71)	1.46(1.06-2.02)	1.19(0.86-1.65)	1.40(1.01-1.94)	0.99(0.71-1.36)
30+	1.25(0.62-2.52)	6.91(4.51-10.57)	1.19(0.83-1.71)	1.05(0.73-1.51)	1.69(1.17-2.42)	2.69(1.85-3.92)	1.43(0.99-2.04)	0.90(0.63-1.30)
**Physical activity (hr/week)***								
0-2.37	1.00 (Reference)	1.00 (Reference)	1.00 (Reference)	1.00 (Reference)	1.00 (Reference)	1.00 (Reference)	1.00 (Reference)	1.00 (Reference)
2.38-5.88	0.81(0.39-1.66)	0.48(0.34-0.67)	0.80(0.57-1.12)	0.75(0.53-1.05)	0.71(0.51-1.00)	0.67(0.48-0.94)	1.02(0.73-1.42)	0.91(0.65-1.28)
5.88+	1.04(0.53-2.05)	0.33(0.24-0.47)	0.82(0.59-1.15)	0.68(0.48-0.95)	0.82(0.59-1.15)	0.73(0.52-1.03)	1.02(0.73-1.43)	1.17(0.84-1.64)
**Rural/Urban residence***								
Rural	1.00 (Reference)	1.00 (Reference)	1.00 (Reference)	1.00 (Reference)	1.00 (Reference)	1.00 (Reference)	1.00 (Reference)	1.00 (Reference)
Town	2.41(0.2-28.96)	2.23(0.60-8.28)	1.50(0.43-5.25)	1.12(0.32-3.89)	1.07(0.30-3.81)	2.16(0.53-8.85)	0.87(0.24-3.08)	1.55(0.42-5.76)
City	1.93(0.34-10.97)	0.94(0.45-1.97)	1.55(0.74-3.26)	1.08(0.52-2.25)	1.90(0.90-4.01)	0.57(0.27-1.19)	0.68(0.32-1.44)	0.70(0.33-1.46)
Suburban	1.87(0.44-7.95)	1.11(0.64-1.93)	1.54(0.88-2.71)	1.13(0.65-1.97)	1.49(0.85-2.62)	0.69(0.39-1.20)	0.61(0.35-1.08)	0.77(0.44-1.35)
Urban	1.37(0.26-7.28)	1.16(0.60-2.24)	1.39(0.71-2.7)	0.99(0.51-1.91)	1.56(0.80-3.03)	0.78(0.40-1.51)	0.49(0.25-0.95)	0.66(0.34-1.28)
**NSAID use***								
None or 1/week	1.00 (Reference)	1.00 (Reference)	1.00 (Reference)	1.00 (Reference)	1.00 (Reference)	1.00 (Reference)	1.00 (Reference)	1.00 (Reference)
>1/week	1.27(0.7-2.31)	1.37(1.03-1.81)	1.02(0.77-1.35)	1.19(0.90-1.57)	1.32(1.00-1.75)	1.30(0.98-1.71)	0.99(0.75-1.31)	1.19(0.90-1.58)
**Statin use***								
None	1.00 (Reference)	1.00 (Reference)	1.00 (Reference)	1.00 (Reference)	1.00 (Reference)	1.00 (Reference)	1.00 (Reference)	1.00 (Reference)
>1/week	0.97(0.51-1.86)	2.08(1.51-2.87)	2.14(1.55-2.95)	1.48(1.08-2.03)	1.36(1.00-1.86)	1.56(1.14-2.14)	1.12(0.82-1.53)	1.30(0.95-1.78)

*Sample sizes for some of the covariates are not 813 due to unknown covariate measures. “N/A”=could not calculate odds ratios or 95% confidence intervals due to sample size for the immune marker.
